# Thai Universal Health Care Coverage scheme promotes the accessibility to cleft lip/palate treatment: the result of cleft care provision assessment using modified Geographic Information System

**DOI:** 10.1186/s12913-022-07784-y

**Published:** 2022-03-29

**Authors:** Wannapong Chonnapasatid, Nita Viwattanatipa, Somchai Manopatanakul, Somchit Jaruratanasirikul

**Affiliations:** 1 Samutprakan Provincial Public Health Office, Mueang Samutprakan District, 20 Phoem Toem 2 Alley, Samutprakan, 10270 Thailand; 2 Department of Orthodontics, Faculty of Dentistry, Mahidol University, 6 Yothi Street, Ratchthewi, Bangkok, 10400 Thailand; 3 Department of Advanced General Dentistry, Faculty of Dentistry, Mahidol University, 6 Yothi Street, Ratchthewi, Bangkok, 10400 Thailand; 4 Department of Pediatrics, Faculty of Medicine, Prince of Songkla University, Songkhla, Thailand

**Keywords:** Cleft Lip, Cleft Palate, Assessment, Resource allocation, Thailand

## Abstract

**Background:**

This study assessed the cleft lip/palate (CL/P) healthcare provision using data from the Thailand National Health Security Office from fiscal years 2012–2016.

**Methods:**

Four national databases of Thailand comprising 1) admitted patient visit, 2) non-admitted patient visit, 3) birth defects registry and 4) civil registration databases were analyzed. All duplicate records were removed by a matching process using national identity number and date of birth prior to data extraction. Modified Geographic Information System was also used to compare each provincial patients with CL/P of Thailand to the number of provincial live births with CL/P.

**Results:**

The results showed that the number of live births with CL/P during this period was 7,775 cases (1,555 cases/fiscal year). While the number of cases with CL/P registered under the Universal Health Care Coverage with hospital stay was 6,715 (86.37%), 927 cases (11.92%) visited hospitals without a stay, and the remaining 133 cases (1.71%) never visited any hospital. Modified Geographic Information System result showed that the provincial CL/P healthcare was relatively well-balanced with the provincial live births with CL/P (r = 0.92, *p* < 0.05). Moreover, provinces with CL/P tertiary care centers attracted more patients from the surrounding provinces.

**Conclusion:**

This study showed that the percentage of patients with CL/P receiving hospital treatment was 98. The Thai Universal Health Care Coverage scheme has promoted the accessibility to CL/P treatment. In order to achieve the best possible comprehensive cleft care coverage, periodical assessment and improvement of the function and accuracy of the national database registry are recommended.

**Supplementary Information:**

The online version contains supplementary material available at 10.1186/s12913-022-07784-y.

## Background

Cleft lip and palate (CL/P) care administration has posed a global health challenge for every country. While most high-income countries cover more than 90 percent of cases and their current policies emphasize improving the quality of care, [1–6] low- and middle-income countries strive to strategize the coverage of registries and treatment for patients with CL/P [7]. It is more demanding to establish efficient cleft care for low- and middle-income countries with more than 1,000 newborns with CL/P per year [8–11].

As children born with CL/P require extensive and usually lifelong management, [12] the quality of received care is highly dependent upon the travelling distance from a cleft center [9–13]. Cleft care should not further burden the patients especially in the low- and middle-income countries; thus, this highlights the importance of reasonably planned government aid [14]. A comprehensive system with cooperation between clinical and clerical work forces is necessary to ensure optimal resource allocation [15–17]. Periodical evaluation of the cleft registries and declaration of resources are reasonable measures to warrant accurate and updated data. Consequently, this measure facilitates the assessment leading to the continuous development of the CL/P care system [1, 18, 19].

To promote cleft care coverage, the Thai Red Cross supports the traveling expenses for the patient’s guardians for every hospital visit. For more than 15 years, the Thai Red Cross has paid approximately 17 USD for every non-admitted visit and 35 USD for an admitted visit to every patient regardless of nationality. In Thailand, this amount of money is more than enough to cover a round-trip by bus for short inter-provincial traveling expenses.

To be in line with the global campaign of systematic tracking of cases with CL/P, the Thailand Birth Defects Registry (BDR) had been advocated and successfully founded in 2010, with collaboration between the Queen Sirikit National Institute of Child Health (QSNICH), Ministry of Public Health (MOPH) and National Health Security Office (NHSO). This project intended to implement the systematic cases with CL/P registration by provincial recordkeeping officers [20, 21]. Simultaneously, the government assigned the NHSO to autonomously channel a major portion of expenses for all cases with CL/P through the Universal Health Care Coverage scheme (UC). Therefore, the NHSO holds access to the nationwide database of every hospital visit of almost every patient with CL/P in Thailand.

 For every patient to receive the correct funding and reimbursement, each hospital visit must be registered using the patient’s own Thai National Identity Number (NIN), issued to every Thai citizen at birth by the Civil Registration Office of the Ministry of Interior [22, 23] This ensures the accuracy of data input and allows the information to be verified.

As the national cleft registry including cleft care coverage and its distributions, are essential and NHSO officially has access to all related databases, it is possible to evaluate the coverage and resource allocation in the CL/P healthcare delivery system. Therefore, this study aimed to assess the provision of care for newborn children with a cleft in Thailand based on the NHSO data.

## Methods

### Research setting

Data from the Thai government's national budget bill for fiscal years 2012–2016 (1^st^ October 2011–30^th^ September 2016) were retrieved from the NHSO after this study was submitted and approved by the Faculty of Dentistry/Pharmacy, Mahidol University Institutional Review Board (COE.No.MU-DT/PY-IRB 2021/015.0706).

### Study population

All newborn children with a cleft in the fiscal years 2012–2016 with complete data of NIN and date of birth (DoB) were included. Exclusion criteria were termination of pregnancy and stillbirth. The NHSO database of cases with CL/P consisted of four main parts, which were hospital visits with or without admission, BDR, and civil registration information.

### Admitted and non-admitted patient visits

Every hospital visit with ICD-10 code Q35-Q37 (World Health Organization International Classification of Diseases, 10^th^ revision) [24] was categorized into admitted (APV) and non-admitted patient visits (NAPV; Table 1). These entries included all hospitals countrywide, comprising regional, general community, and private hospitals. APV provided more detailed information, such as the number and duration of each admission. Besides, the number and percentage of cases with CL/P classified by cleft type were also available.

**Table 1:** Data retrieved from the National Health Security Office (NHSO)

	NIN	DoB	Province	Thai nationality	Live birth	Admission visit	ICD-10 code
Civil registration	O	O	O	O	O		
APV	O	O	O		O	O	O
NAPV	O	O	O		O		O
BDR	O	O			O		O

*Abbreviations*: *APV* Admitted patient visits, *BDR* Birth defects registry, *NAPV* Non-admitted patient visits, *NIN* National identity number, *DoB* Date of birth, *O* Data recorded in database

### Birth defects registry (BDR)

BDR showed all babies coded as ICD-10 Q35-Q37 indicating CL/P. These babies with CL/P were registered at the hospital where they were born.

### Civil registration

The civil registration system of the Ministry of Interior legally registers every Thai newborn with a thirteen-digit code as the NIN. When this NIN is matched to DoB and data from the NHSO, it aids in identity verification and removal of all duplicate records from these four databases. It also enables the tracking of the number of provincial live births. The details of each database are displayed in Table 1.

### Coverage and resource allocation assessment

All CL/P records from all databases were sorted and verified by two researchers (WC and SM), then re-analyzed and reported with regards to the evaluation of comprehensive cleft care coverage. To determine the provincial balance of care, the correlation between the log values of the provincial number of live births with CL/P and the number of admitted patients was also calculated.

### Geographic Information system (GIS)

To assess the provincial balance of care, a modified Geographic Information System (GIS) for visual element mapping was utilized to ease the understanding of the spatial distribution of the data [25–27]. The map of Thailand was divided into five regions as follows: 1) Northern region (17 provinces), 2) Central region (16 provinces), 3) Northeastern region (20 provinces), 4) Eastern region (9 provinces), and 5) Southern region (14 provinces). [Fig. 4 for central part. For figures of all parts of Thailand, please see the supplementary section. It also provides the link (https://dt.mahidol.ac.th/en/department-of-advanced-general-dentistry/research/gis_cleft/) for the enlarged mappings.]

The associated CL/P data including the number of patients with CL/P, were grouped according to provinces. These data were prepared in a spreadsheet which comprised two main parameters, namely: 1) the number of patients with CL/P admitted to the hospital (APV) and 2) the number of live births with CL/P.

These data sets were then mathematically translated into circular graphics where the relative sizes of each circle with specific radii corresponded with the aforementioned number of patients with CL/P. The method used to generate these circular graphics is as follows:

- Step 1: The number of patients with CL/P = area of a circle = π r^2^
- Step 2: Radius (r) was determined to construct the circular graphics for each province
- Step 3: Geographic map of Thailand was imported into Microsoft PowerPoint with controlled scale
- Step 4: The circular graphics were drawn using Microsoft PowerPoint, where the radius of each circle was specified using the ‘size & position’ command according to the calculated values from Step 2
- Step 5: The circular graphics were superimposed over the figures of each geographic region of Thailand

## Results

### Number of cases and visits identified by sources

#### APV database

**Fig. 1:**
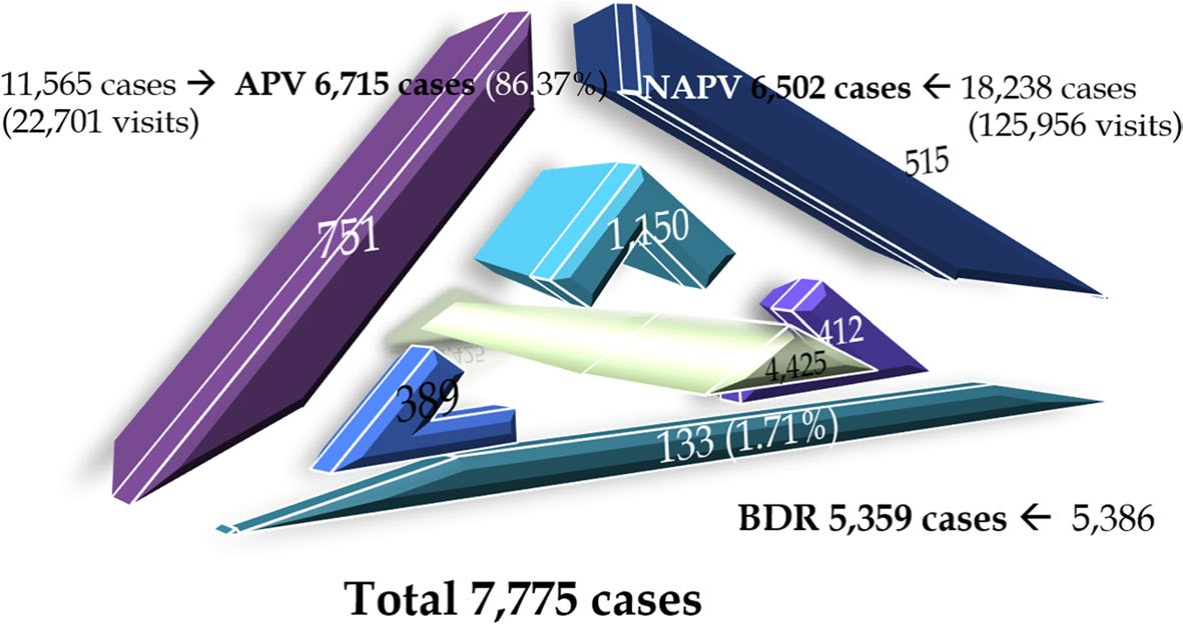
National identity number (NIN) and date of birth (DoB) from the civil registration enabled the removal of replicate records among the three databases [admitted patient visit (APV), non-admitted patient visit (NAPV), birth defects registry (BDR)]. After verification, the total number of new cases with CL/P was 7,775 during the fiscal years of 2012–2016. A proportionate three-dimensional diagram depicting the number of cases was constructed. The outermost parts show three databases where cases appeared in one database only. The middle layer shows cross-validation using two databases. The innermost triangle signifies the cases captured by all three databases, which show more than half of the total count (56.91%; 4,425 out of 7,775). APV of 6,715 cases demonstrated comprehensive cleft care coverage of 86.37%. The 133 cases (1.71%) recorded solely in the BDR indicate that these patients had never visited the hospitals. The remaining proportion of patients with CL/P who had visited the hospitals at least once was 98.29% or 7,642 out of 7,775 cases

After the exclusion criteria were applied, a total of 15,757 visits by 6,715 admitted patients from a grand total of 22,701 admitted visits by 11,565 recorded patients were obtained (Fig. 1). From the 6,715 admitted cases, the proportion of patients with cleft lip (CL), cleft palate (CP), and cleft lip with cleft palate (CLP) were 23%, 27% and 50%, respectively. A total of 25% of all cases were patients with CL/P as a non-isolated anomaly (coexisting with other congenital anomalies). The detailed breakdown of cases according to type is summarized in Table 2.

**Table 2:** Statistics of cases with CL/P classified by cleft type and presence of coexisting congenital anomalies from the APV database

Type of cleft	CL		CP		CLP		Total	
	N	%	N	%	N	%	N	%
Patients with CL/P as an isolated anomaly	1,355	88	1,248	68	2,427	73	5,030	75
Patients with CL/P as a non-isolated anomaly (coexisting with other congenital anomalies)	190	12	595	32	900	27	1,685	25
Total	1,545	23	1,843	27	3,327	50	6,715	

*Abbreviations*: *CL/P* Cleft lip and/or cleft palate = cleft lip + cleft lip and palate + cleft palate, *CL* Cleft lip, *CP* Cleft palate, *CLP* Cleft lip with cleft palate

Upon further analysis of the APV data, most patients (2,229 cases) were admitted to the hospital only once. The highest number of hospital admissions per patient was 20. In addition, four patients required 17, 18, 19, and 20 admitted visits each (Table 3).

**Table 3:** Admission visit per patient from the APV database. The number in the bracket shows the number of visits by cases with a non-isolated cleft (coexisting with other congenital anomalies)

Admission visit(s)/patient	CL	CP	CLP	Number of cases
1	583/644	690/953	380/632	1,653/2,229
	(61)/644	(263)/953	(252)/632	(576)/2,229
2	656/748	415/548	613/761	1,684/2,057
	(92)/748	(133)/548	(148)/761	(373)/2,057
3	99/122	85/165	799/1,008	983/1,295
	(23)/122	(80)/165	(209)/1,008	(312)/1,295
4	7/10	34/68	428/565	469/643
	(3)/10	(34)/68	(137)/565	(174)/643
5	8/14	18/53	150/215	176/282
	(6)/14	(35)/53	(65)/215	(106)/282
6	1/1	5/23	33/73	39/97
	(0)/1	(18)/23	(40)/73	(58)/97
7	1/2	1/10	15/36	17/48
	(1)/2	(9)/10	(21)/36	(31)/48
8	0/2	0/9	8/21	8/32
	(2)/2	(9)/9	(13)/21	(24)/32
9	0/1	0/5	0/3	0/9
	(1)/1	(5)/5	(3)/3	(9)/9
10	0/0	0/2	1/6	1/8
	(0)/0	(2)/2	(5)/6	(7)/8
More than 10	0/1	0/7	0/7	0/15
	(1)/1	(7)/7	(7)/7	(15)/15
Total	1,355/1,545	1,248/1,843	2,427/3,327	5,030/6,715
	(190)/1,545	(595)/1,843	(900)/3,327	(1,685)/6,715

*Abbreviations CL* Cleft lip, *CP* Cleft palate, *CLP* Cleft lip with cleft palate

Regarding the duration of admission, 97% of admissions were less than 1 month. The longest admission was over 12 months at Chonburi province (Table 4). Bangkok recorded the highest number of admissions (1,993 visits) and patients (774 patients), followed by Khon Kaen (1,063 visits, 269 patients) and Nakhon Ratchasima (810 visits, 281 patients). In contrast, Samutsongkhram showed the least number of admission visits (9 visits) and patients (8 patients), followed by Phangnga (20 visits, 17 patients) and Trat (21 visits, 18 patients).

**Table 4:** Duration of admission and the number of cases from the APV database classified as cleft lip (CL), cleft palate (CP), and cleft lip with palate (CLP). Number in bracket shows non-isolated cleft

Duration of Admission	Number of visits			Total number of visits
	CL	CP	CLP	
Less than 3 days	2,425/2,608	1,924/2,275	3,246/3,741	7,595/8,624
	(183)/2,608	(351)/2,275	(495)/3,741	(1,029)/8,624
4–7 days	807/872	1,263/1,503	1,856/2,164	3,926/4,539
	(65)/872	(240)/1,503	(308)/2,164	(613)/4,539
5–11 days	198/228	225/355	424/560	847/1,143
	(30)/228	(130)/355	(136)/560	(296)/1,143
12–14 days	25/35	63/141	115/183	203/359
	(10)/35	(78)/141	(68)/183	(156)/359
15–21 days	30/44	50/149	92/195	172/388
	(14)/44	(99)/149	(103)/195	(216)/388
1 month	14/30	36/93	43/115	93/238
	(16)/30	(57)/93	(72)/115	(145)/238
2 months	12/27	21/146	35/139	68/312
	(15)/27	(125)/146	(104)/139	(244)/312
3 months	2/8	9/37	9/39	20/84
	(6)/8	(28)/37	(30)/39	(64)/84
4 months	1/2	5/18	4/17	10/37
	(1)/2	(13)/18	(13)/17	(27)/37
5 months	0/0	1/4	1/6	2/10
	(0)/0	(3)/4	(5)/6	(8)/10
6 months	0/1	0/7	0/5	0/13
	(1)/1	(7)/7	(5)/5	(13)/13
More than 6 months	0/1	1/5	0/4	1/10
	(1)/1	(4)/5	(4)/4	(9)/10
Total	3,514/3,856	3,598/4,733	5,825/7,168	12,937/15,757
	(342)/3,856	(1,135)/4,733	(1,343)/7,168	(2,820)/15,757

Comparison between the number of patients with CL/P admitted to the hospital versus the number of births with CL/P per year is listed. For provinces with the number of admissions exceeding the number of births with CL/P, Bangkok was in the lead, followed by Khon Kaen, Nakhon Ratchasima, Phitsanulok, and Songkhla. In contrast, Samutprakan showed the highest opposite values (the number of births with CL/P births exceeded the number of admissions) followed by Pathumthani, Samutsakhon, Phetchabun, and Kamphaengphet.

From this data, the coverage of patients with CL/P was also calculated and displayed in percentages. Despite having the lowest number of patients with CL/P, Nakhon Nayok showed the highest coverage (159%), followed by Phitsanulok (143.48%) and Khon Kaen (143.09%). On the other hand, Pathumthani and Samutprakan showed the poorest coverage at 43.48% and 44.16%, respectively.

### NAPV database

After the exclusion criteria were applied to 125,956 NAPVs, the number decreased to 18,238 visits by 6,502 patients (Fig. 1). The significance of this database was for the removal of duplicate registrations compared to the other two databases, the APV and BDR.

### BDR database

From the BDR database, after exclusion of duplicate records, the number of records dropped from 5,386 cases to 5,359 cases.

### Civil registration information

By law, live birth registries record almost all Thai newborns. Unique thirteen-digit NINs are given to every individual newborn. NIN and DoB were used to verify duplicate records for both the BDR and treatment fee settlement (APV and NAPV). This verification method also facilitated the documentation of provincial birth records and enabled the assessment of resource allocation.

Once repeat registrations were removed, the total number of cases with CL/P was 7,775 (Fig. 1), with an average of 1,555 cases per fiscal year (standard deviation = 154.19). A declining trend in the number of cases per year was also observed (Table 5).

**Table 5:** Number of live births with CL/P for the fiscal years of 2012–2016

Fiscal Year	Duration	Number of Cases
2012	1^st^ October 2011–30^th^ September 2012	1,739
2013	1^st^ October 2012–30^th^ September 2013	1,615
2014	1^st^ October 2013–30^th^ September 2014	1,557
2015	1^st^ October 2014–30^th^ September 2015	1,549
2016	1^st^ October 2015–30^th^ September 2016	1,315
2012–2016	1^st^ October 2011–30^th^ September 2016	7,775

### Cleft care provision assessment from multiple sources verification

**Fig. 2:**
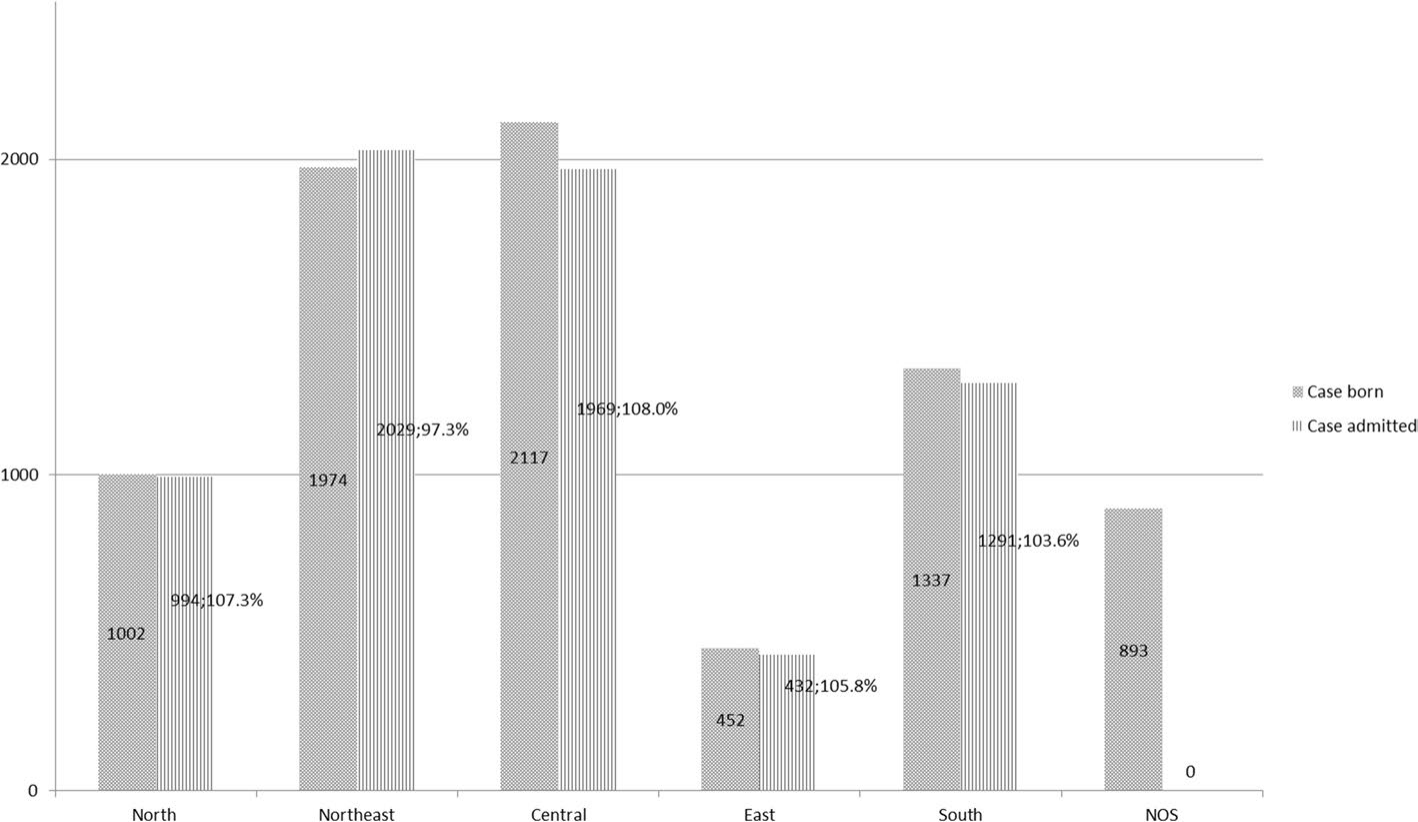
Bar chart depicting the regional distribution of births with CL/P and the number of admissions and coverage percentage. (NOS: not otherwise specified)

Once the records were verified, the ratio of hospital admissions of cases with CL/P to the number of newborn CL/P cases was found to be 6,715:7,775. This number was cross-checked with the provincial case records. However, 893 newborns with CL/P (11.49%) were excluded due to unspecified data, [not otherwise specified (NOS); confusing or incomplete provincial data; Fig. 2], hence, the total number of provincial newborn cases with CL/P was only 6,882.

**Fig. 3:**
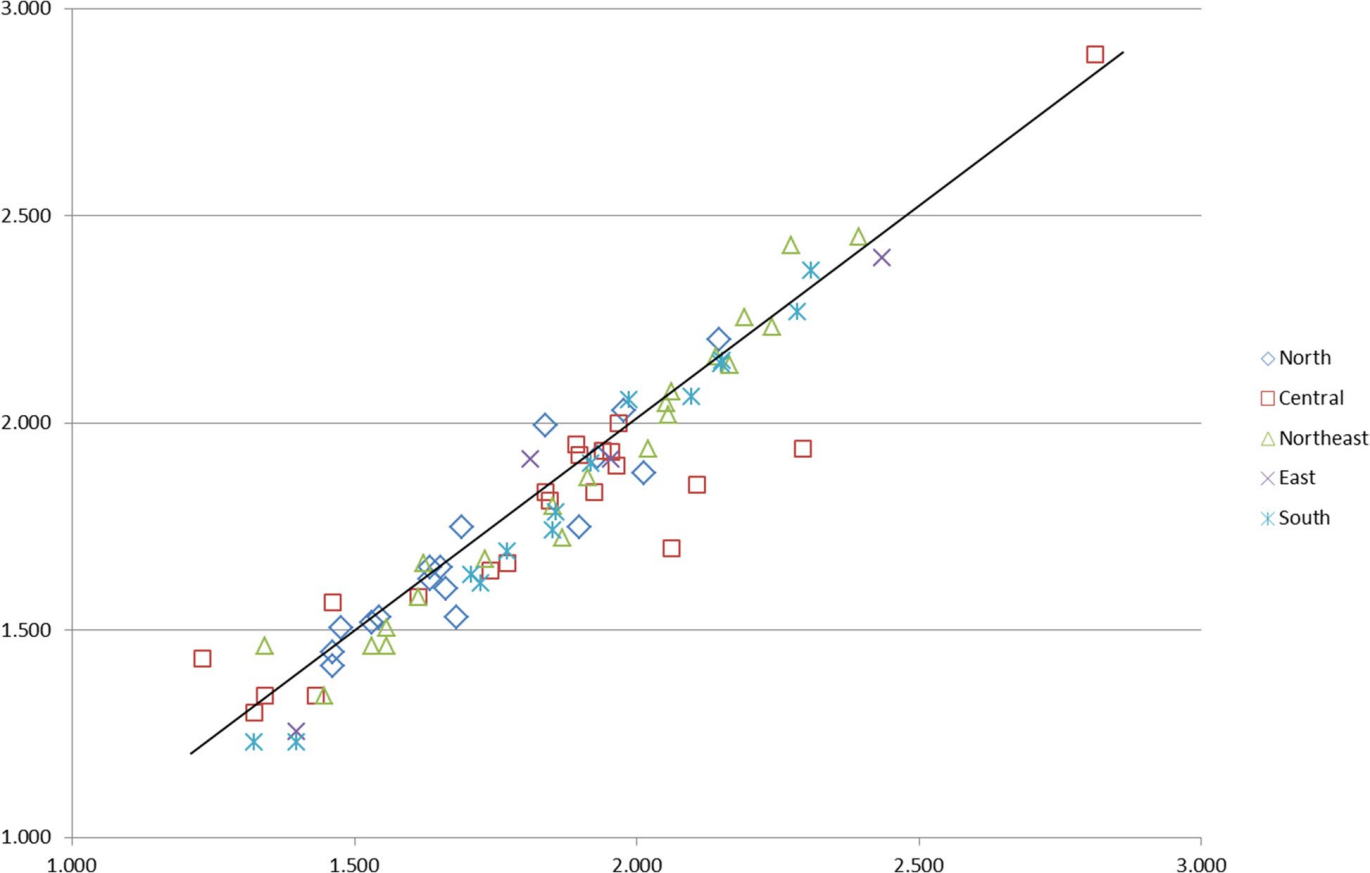
Correlation plot between the log number of newborns with CL/P against the number of CL/P admissions throughout the fiscal years 2012–2016 (r = 0.92; *p* < 0.05)

These numbers were reported in relation to the provision of treatment in each province. The regional and provincial balance between cases and services are displayed in Figs. 2 and 3. The correlation between the provincial log number of live births with CL/P and the number of admissions was high (r = 0.92; *p* < 0.05).

### Cleft care provision assessment illustration using modified GIS mappings

**Fig. 4:**
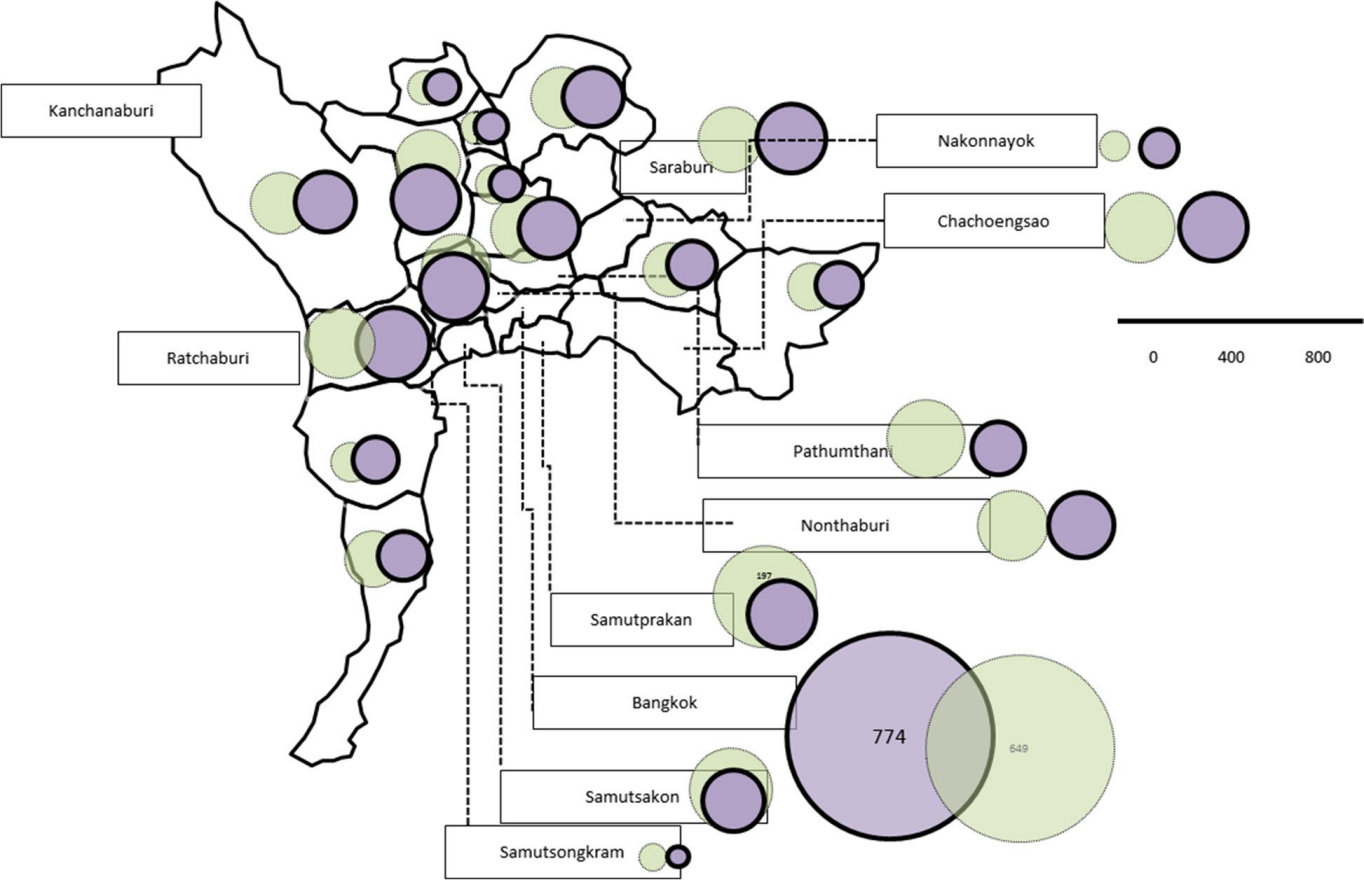
Newborns with CL/P (green circle) compared to CL/P admissions (purple circle) according to provinces in the Central region. The province with university hospitals such as Bangkok showed the largest number of patients and treatment provision. It can be inferred that the suburban areas around provinces with university hospitals contributed to the capital city's high numbers. Other regions of Thailand show the same tendency, and the figures are shown in the supplementary information section below. The circle diameter represents the number of patients measured from the linear scale. This map was edited from https://yourfreetemplates.com/free-thailand-editable-map/ using Microsoft PowerPoint

A provincial comparison of 5-fiscal-year admissions and live births with CL/P in various country regions is presented. Figure 4 shows the central part of Thailand, and the link in the supplementary information section shows all other regions using modified GIS mappings. A moderate balance between admissions and births can be seen in almost all regions.

## Discussion

Complete coverage of all cases with CL/P is one of the ultimate goals of worldwide healthcare providers [15]. It is more challenging to optimize and sustain cleft care in low- and middle-income countries, especially with more than a thousand newborns with CL/P each year [7, 28] The results of our study produced a total of 7,775 cases over a period of 60 months. On average, the number of new cases was 1,555 every 12 months (standard deviation = 154.19), with a declining trend of new cases.

The percentage of cases who visited hospitals at least once was high at 98.29%, while the number of admissions was 6,715 cases (86.37% coverage) with 15,757 visits. This 86.37% figure implies a moderate goal achievement in terms of comprehensive case coverage in Thailand. Continuous attempts to increase the case coverage and hospital visits will be carried out to be promoted to reach a high goal achievement of 90%. In Thailand’s attempt to maximize cleft care coverage, persistent efforts have been made to judiciously distribute resources for the benefit of all cases. The results of this study have demonstrated that Thailand has above average cleft care coverage despite being a middle-income developing country.

This study included only patients with CL/P who were less than 5 years of age. Within this age range, complex cases require many forms of multi-disciplinary management. Patients of this age group also fully rely on a guardian for transportation. Through efforts for quality improvement, one of the goals has been to establish cleft centers that are easily and readily accessible to handle essential, complex, or urgent specialist care [25]. For these reasons, this study focused on assessing the optimal resource allocation for this age group that truly needs comprehensive support. It also should be noted here that apart from the incurred treatment cost supported by the NHSO, the Thai Red Cross supports the travelling expenses for the patient’s guardians for every hospital visit. This support acts as an unseen helping hand for this success.

From our results, we can infer that the regional distribution of service provision for patients with CL/P has been well balanced. (Fig. 3) From the statistical analysis and mapping, it can be observed that most circles in each province were moderately well proportioned [Figs. 2, 3 and 4 and the link in the supplementary information section]. The number of patients was more noticeably centered around cleft centers and university hospitals strategically set up in each country region. It can be assumed that cleft centers and university hospitals could account for more patients and longer duration of hospital admission. Besides, these centers likely receive a significant number of patients from the surrounding provinces. This would explain why the adjacent provinces’ hospitals recorded lower numbers of admissions even though the overall coverage was relatively well balanced.

### Strengths

The strengths of this study can be explained as follows. First, it assesses the coverage of cleft care in Thailand via using several databases. Further, the long-term, national-level data extraction was carried out using a novel capturing and cross-verification method using DoB and the by-law Thai NIN system. As a result, child with cleft was counted only once no matter how often the baby visits the hospital or how different of the regional cleft care protocols are. In the near future, the data completeness will be further enhanced, especially when the Thai NIN is adapted into a digital format [29]. The details of this methodology have previously been reported in an earlier study [11]. In addition, the number and duration of visits by cases with a non-isolated cleft which require more government statement of expenditure were distinctly revealed. The Modified GIS used in this study is also simple, economical and can be readily available even in developing countries. The result, however, is interesting and can contribute to useful information especially for national policy makers.

### Limitations

However, it should be noted that these mappings and tabulations (Figs. 2, 3 and 4 and link in the supplementary information section) employed data with missing information. A total of 893 cases of newborns with CLP had incomplete or unclear data. Though these NOS cases reveal the limitations of this study and registration system in Thailand, this figure was still less than the number of patients who had never visited a doctor and were never admitted to the hospital (1,060 cases). Another drawback of our study was that secondary data sources were analyzed using the ICD-10 classification. This would cause ambiguity in distinguishing the number of cases with syndromic cleft versus non-isolated cleft. In addition, the data obtained for this study is not the most recent, as only records up to the year 2016 were included. Different protocol among different cleft centers and different timing of referral also affects the number of visits and care pathways on attendances. Conversely, clerical staff training and improvement of data input technology and networking should mitigate the weaknesses of this study. In addition, active case finding with clinical diagnosis validation was suggested to improve the completeness of the data. Finally, future research should take all these shortcomings to add to and improve upon the gaps in knowledge.

## Conclusions

- The percentage of Thai patients with CL/P (newborns up to 5 years) registered into hospital healthcare services under UC was 98.29%.
- The percentage of Thai patients with CL/P under UC that have visited a hospital with admission was 86.37% and non-admission was 11.92%.
- The percentage of Thai patients with CL/P who have never visited a hospital was 1.71%.
- According to the statistical and modified GIS analyses, the provincial hospital healthcare services were relatively well-balanced with the provincial live births with CL/P.
- CL/P tertiary care centers attracted more patients and provided more treatment than other hospital categories.
- Thailand’s UC governmental scheme has successfully promoted treatment coverage for patients with CL/P.

## Supplementary Information

**Additional file 1.**

## Data Availability

The data that support the findings of this study are available from NHSO but restrictions apply to the availability of these data, which were used under license for the current study, and so are not publicly available. Data are however available from the corresponding authors (Somchai Manopatanakul, msomchai@rocketmail.com) upon reasonable request and with permission of NHSO.

## Abbreviations

APV: Admitted patient visits
BDR: Birth defect registration
CL: Cleft lip
CL/P: Cleft lip and palate
CLP: Cleft lip with cleft palate
CP: Cleft palate
DoB: Date of birth
GIS: Geographic Information System
ICD-10: World Health Organization International Classification of Diseases (10th revision)
MOPH: The Ministry of Public Health
NAPV: Non-admitted patient visits
NHSO: National Health Security Office
NIN: National identity number
NOS: Not otherwise specified
QSNICH: Queen Sirikit National Institute of Child Health
UC: Universal health care coverage scheme
