# Supplementary material for: Thai Universal Health Care Coverage scheme promotes the accessibility to cleft lip/palate treatment: the result of cleft care provision assessment using modified Geographic Information System
Source: BMC Health Serv Res. 2022 Mar 29;22:416. doi: 10.1186/s12913-022-07784-y (PMC8966314; doi:10.1186/s12913-022-07784-y)
Supplement: Supplementary file 1 — Additional file 1. [file 12913_2022_7784_MOESM1_ESM.docx]

**A modified Geographic Information System (GIS): spatial distribution of cleft care provision for 5 regions of Thailand.**

**Please follow this link below for more information with enlarged visual element mappings.** <https://dt.mahidol.ac.th/en/department-of-advanced-general-dentistry/research/gis_cleft/>

The map of Thailand was divided into five regions (Figures 1-5) as follows: 1) Northern region (17 provinces), 2) Central region (16 provinces), 3) Northeastern region (20 provinces), 4) Eastern region (9 provinces), and 5) Southern region (14 provinces).

**
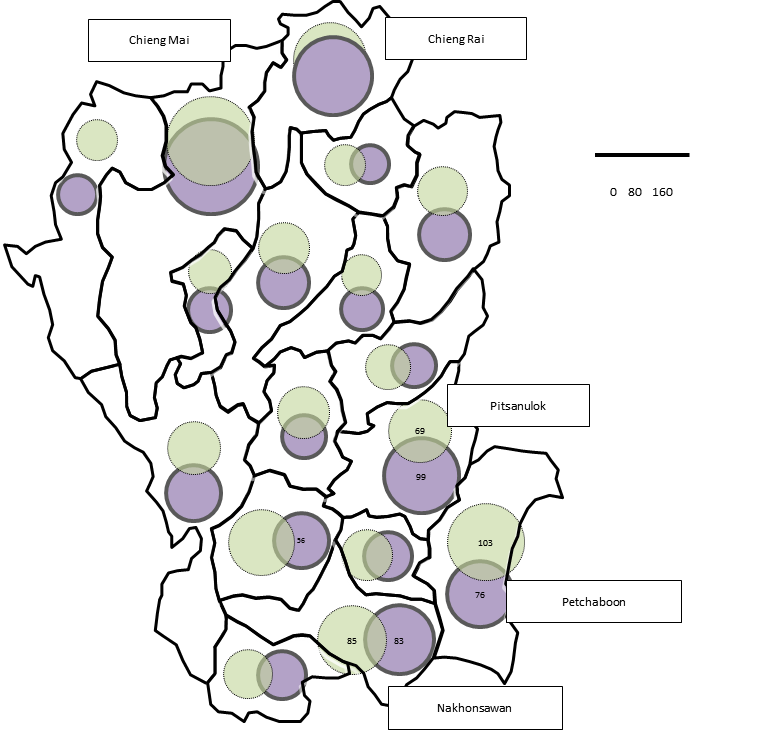
**

**Figure 1:** Newborns with CL/P (green circle) compared to CL/P admissions (purple circle) according to provinces in the Northern region. Chieng Mai province with a university hospital showed the largest number of patients and treatment provision. The circle diameter represents the number of patients as can be measured from the linear scale.


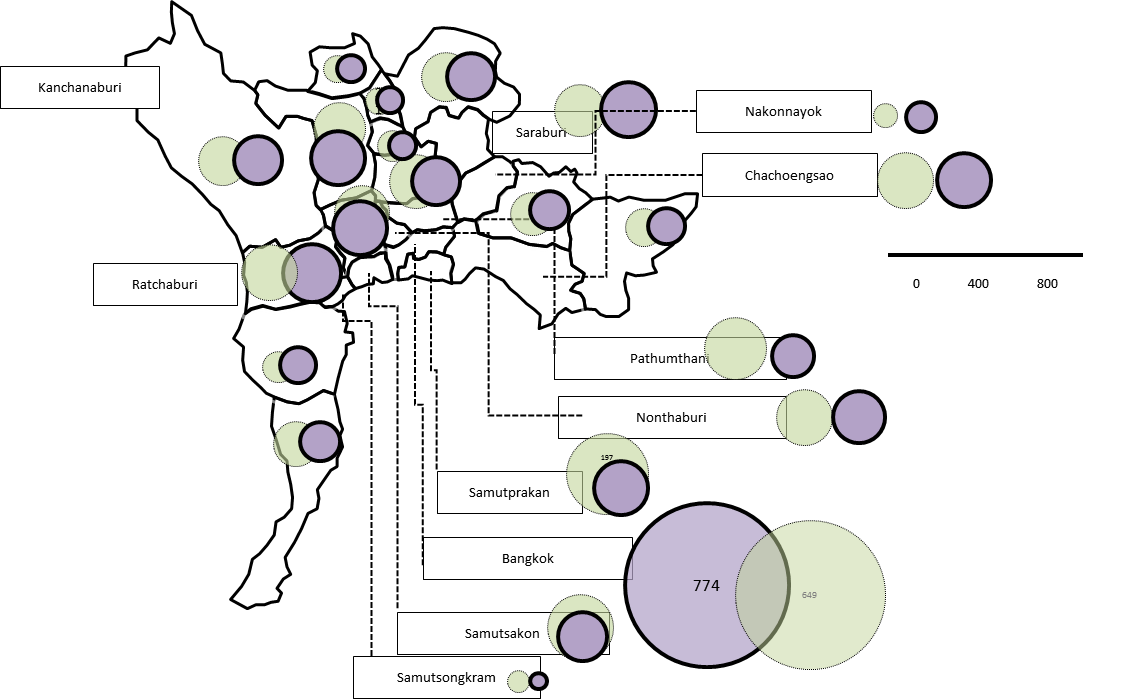


**Figure 2:** Newborns with CL/P (green circle) compared to CL/P admissions (purple circle) according to provinces in the Central region. The province with university hospitals such as Bangkok showed the largest number of patients and treatment provision. It can be inferred that the suburban areas around provinces with university hospitals contributed to the capital city's high numbers.


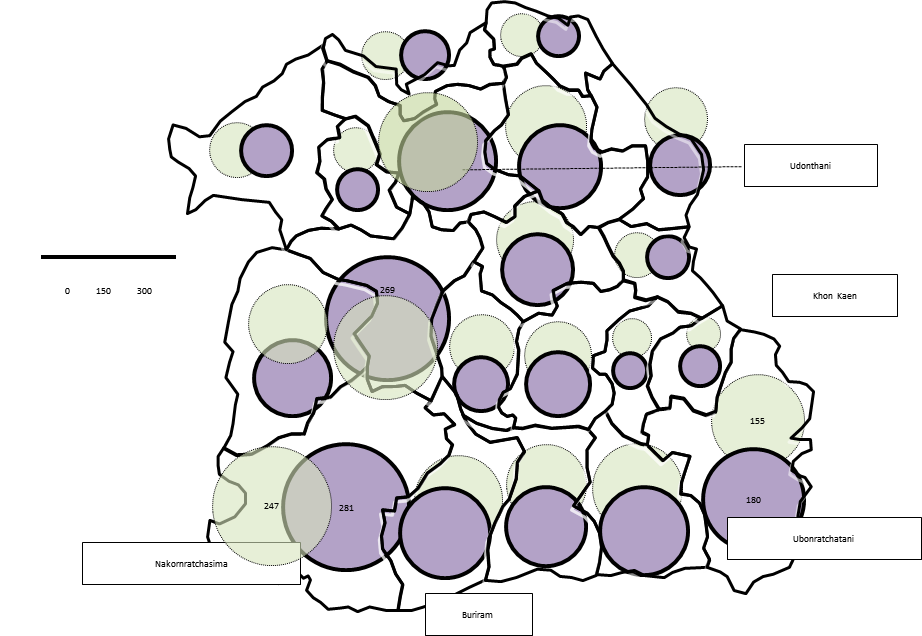


**Figure 3:** Newborns with CL/P (green circle) compared to CL/P admissions (purple circle) according to provinces in the Northeastern region. Nakhon Ratchasima and Khon Kaen provinces which have active cleft centers showed the largest number of patients and treatment provision.


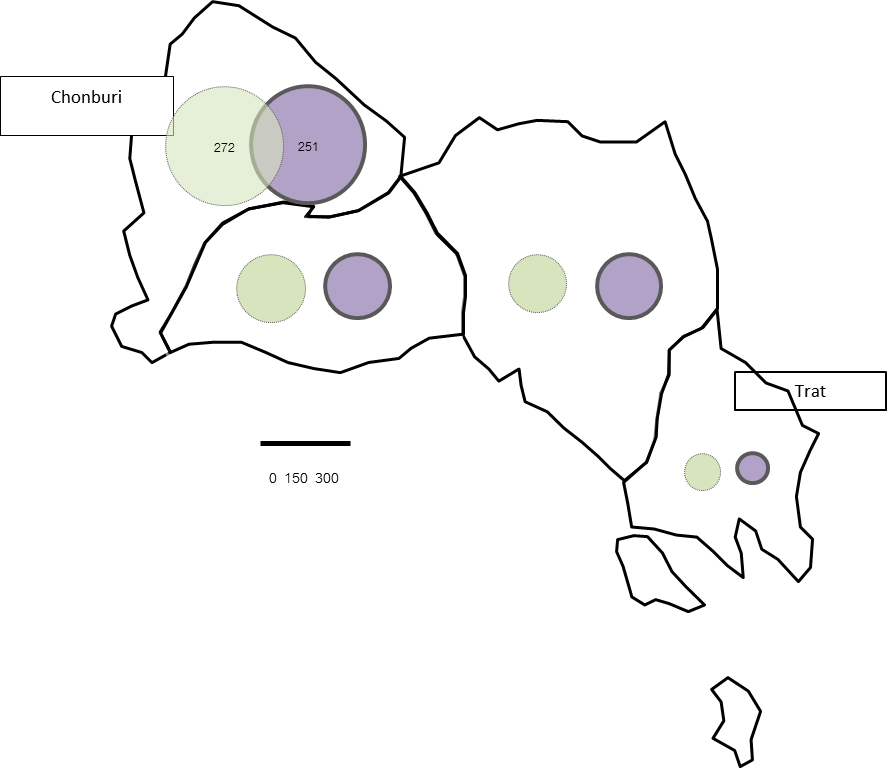


**Figure 4:** Newborns with CL/P (green circle) compared to CL/P admissions (purple circle) according to provinces in the Eastern region. Chonburi province showed the largest number of patients and treatment provision.

**
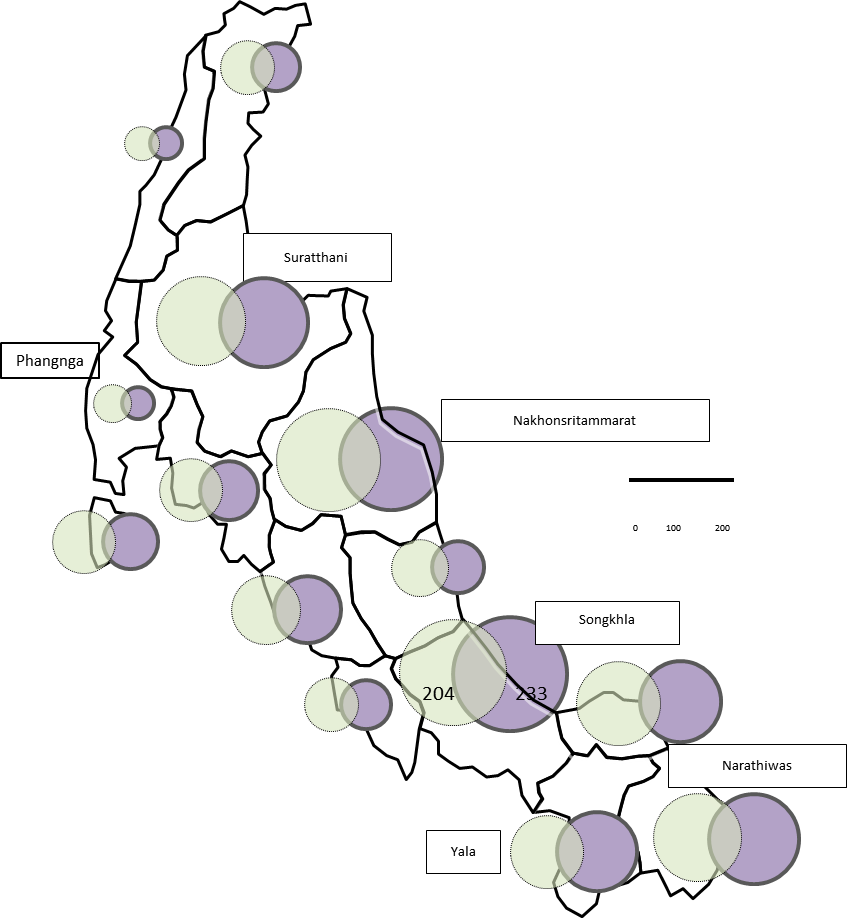
**

**Figure 5:** Newborns with CL/P (green circle) compared to CL/P admissions (purple circle) according to provinces in the Southern region. Likewise, Songkhla province with a university hospital showed the largest number of patients and treatment provision.

It can be inferred that the regional distribution of service provision for patients with CL/P has been well balanced. From the mapping, it can be observed that most circles in each province were moderately well proportioned. The number of patients was more noticeably centered on cleft centers and university hospitals that are strategically set up in each region of the country. (All maps were edited from https://yourfreetemplates.com/free-thailand-editable-map/using Microsoft PowerPoint.)
